# Non-viral gene therapy for hemophilia A: long-term outcomes of minicircle FVIII delivery in a mouse model

**DOI:** 10.3389/fphar.2026.1742144

**Published:** 2026-04-09

**Authors:** Yung-Tsung Kao, Chih-Ching Yen, Gary Ro-Lin Chang, Hsin-Tse Chiang, Jen-Kun Chen, Muhammad Sufian, I.-Chien Chen, Shang-Hsun Yang, Chuan-Mu Chen

**Affiliations:** 1 Department of Life Sciences, and Doctorial Program in Translational Medicine, National Chung Hsing University, Taichung, Taiwan; 2 Ph.D. Program in Tissue Engineering and Regenerative Medicine, National Health Research Institutes and National Chung Hsing University, Taichung, Taiwan; 3 Department of Internal Medicine, China Medical University Hospital, and China Medical University, Taichung, Taiwan; 4 Institute of Biomedical Engineering and Nanomedicine, National Health Research Institutes, Tainan, Taiwan; 5 Institute of Molecular Biology and Biotechnology (IMBB), The University of Lahore, Lahore, Pakistan; 6 Department of Physiology, Institute of Basic Medical Sciences, National Cheng Kung University, Tainan, Taiwan; 7 The iEGG and Animal Biotechnology Center, Rong Hsing Research Center for Translational Medicine, National Chung Hsing University, Taichung, Taiwan; 8 Center for General Educational, National Quemoy University, Kinmen, Taiwan

**Keywords:** coagulation factor VIII (FVIII), E1984V point mutation, hemophilia A, mini-vector, non-viral gene therapy

## Abstract

Hemophilia A, an X-linked bleeding disorder caused by factor VIII (FVIII) deficiency, necessitates lifelong factor replacement therapy with high treatment burden. To explore a non-viral alternative, we evaluated minicircle DNA carrying the FVIII-E1984V mutation—engineered for improved stability and activity—as a gene therapy for sustained coagulation correction. Mini-circle DNA constructs (with the bacterial backbone excised) encoding either wild-type FVIII or E1984V-FVIII were delivered *via* hydrodynamic tail vein injection into FVIII-KO mice, a typical murine model of hemophilia A. Coagulation outcomes (aPTT, FVIII activity, tail-clip assay) and transgene persistence were monitored over 26 weeks. Minicircle DNA delivery demonstrated higher transfection efficiency *in vitro* and sustained coagulation improvement *in vivo* for at least 26 weeks, markedly exceeding the short durability of conventional FVIII infusion (days *versus* weeks). Furthermore, exogenous FVIII DNA and RNA persisted in hepatocytes without evidence of hepatotoxicity. These findings highlight minicircle DNA–based FVIII gene therapy as a promising strategy for hemophilia A. Future studies will focus on optimizing vector design for sustained expression, advancing toward clinical translation.

## Introduction

Hemophilia A (HA) is an X-linked recessive bleeding disorder caused by deficiency of coagulation factor VIII (FVIII), predominantly affecting males (Chowdary et al., 2025). Rising global prevalence sees patients enduring spontaneous joint/muscle hemorrhages and bruising, imposing significant quality-of-life burdens (Yen et al., 2022; Stephensen et al., 2009). Standard FVIII replacement therapy is hampered by short half-life, necessitating frequent infusions that create substantial treatment burdens (Verhagen et al., 2024; Hodroj et al., 2022).

Gene therapy offers long-term therapeutic potential for hemophilia A through single-dose delivery of functional FVIII transgenes (Pipe et al., 2022; Perrin et al., 2019). While adeno-associated virus (AAV) vectors dominate clinical applications—with recent approvals of Roctavian® (hemophilia A) and Hemgenix® (hemophilia B)—viral approaches face significant limitations (Samelson-Jones and George, 2023; Leebeek and Miesbach, 2021; George, 2021). Immune responses against AAV capsids remain a major barrier, including complement activation triggering thrombotic microangiopathy or hepatotoxicity (Samelson-Jones et al., 2024; Navale et al., 2022) and pre-existing antibodies that neutralize vectors and amplify innate immune reactions *via* Toll-like receptor 9 (TLR9) pathways (Kachanov et al., 2024; Hamilton and Wright, 2021). Additionally, AAV’s packaging capacity (∼4.7 kb) severely con-strains delivery of large transgenes like full-length FVIII (7 kb), reducing expression efficiency. Recently, emicizumab, a bispecific monoclonal antibody bridging activated factor IX and factor X, has emerged as a novel prophylactic treatment for hemophilia A. It can be administered subcutaneously and is effective in patients with or without inhibitors, reducing bleeding frequency compared to conventional FVIII replacement therapy (Oldenburg et al., 2017).

Non-viral strategies circumvent viral immunogenicity and accommodate larger pay-loads, but introduce distinct challenges (Zu and Gao, 2021; Ramamoorth and Narvekar, 2015). Conventional plasmids risk genomic integration events and carry bacterial backbone elements (e.g., antibiotic resistance genes, origins of replication) that provoke inflammatory responses (Williams and Paez, 2023; Vandermeulen et al., 2011; Chen et al., 2023). Minicircle technology eliminates these immunogenic sequences through site-specific recombination (Kay et al., 2010), while emerging platforms like ultrasound-mediated delivery enable organ-specific targeting without viral components (Arevalo-Soliz et al., 2020). Nevertheless, DNA fragmentation during non-viral delivery still poses genotoxicity risks through random integration (Wang et al., 2023).

To overcome the limitations of conventional plasmid vectors and reduce immunogenicity, this study utilizes minicircle DNA technology, which eliminates bacterial back-bone sequences such as antibiotic resistance genes and origins of replication through arabinose-induced site-specific recombination in *E. coli* ZYCY10P3S2T competent cells (Kay et al., 2010; Arevalo-Soliz et al., 2020). The removal of these bacterial elements is crucial, as residual sequences—particularly CpG motifs—can activate TLR9 and stimulate host inflammatory responses, thereby compromising gene therapy efficacy and safety (Williams and Paez, 2023; Vandermeulen et al., 2011; Chen et al., 2023). In addition, to enhance the therapeutic potential of the delivered FVIII gene, we introduce the E1984V mutation (glutamate to valine substitution at position 1984), which has been shown to retain over 80% of wild-type specific activity, increase thermostability approxi-mately 2-fold, and enhance FVIII stability by four to eight fold and prolong its plasma half-life, reducing the frequency of dosing required for effective treatment (Wakabayashi et al., 2008).

This study aims to evaluate the therapeutic potential of FVIII minicircle DNA plasmids delivered *via* tail vein injection in a murine model of hemophilia A over a 26-week period. Coagulation restoration will be assessed through serial tail-clip assays, a validated method for quantifying bleeding phenotypes in hemophilia models, alongside FVIII activity kinetics measured by chromogenic assays. Post-sacrifice analyses will include liver-specific mapping of exogenous FVIII distribution by immunohistochemistry and PCR. In addition, comprehensive histopathological screening using H&E staining will be employed to examine inflammation, while hepatotoxicity will be evaluated through liver index measurements (ALT, AST, and ALKP) to detect potential DNA fragmentation–related adverse effects tissue analysis will include liver mapping of exogenous FVIII distribution *via* immunohistochemistry and PCR, while comprehensive histopathological screening will assess inflammation and potential hepatotoxicity from DNA fragmentation through liver index (ALT, AST and ALKP).

## Materials and methods

### Animals

Hemophilia A model mice (FVIII-knockout strain: B6; 129S-F8^tm1kaz/J^) and wild-type controls (129S1/SvImJ) were obtained from The Jackson Laboratory (Bar Harbor, ME, USA) and BioLASCO Taiwan Co., Ltd. (Taipei, Taiwan), respectively. All animals were housed under specific pathogen-free conditions with a 12-h light/dark cycle, controlled temperature (22 °C ± 1 °C), and *ad libitum* access to food/water. Experimental procedures complied with the National Institutes of Health *Guide for the Care and Use of Laboratory Animals* (eighth edition) and were approved by the National Chung Hsing University Institutional Animal Care and Use Committee (IACUC Protocol #104-045).

### Preparation of minicircle DNA vector

The B-domain deleted (BDD) FVIII cDNA was cloned into the pMC.BESPX minicircle vector (MN100B, System Biosciences, Palo Alto, CA, USA), incorporating an EGFP-T2A reporter, and site-directed mutagenesis introduced the E1984V mutation using primers: forward 5′-CTC​TAT​CCA​GGT​GTT​TTT​GTG​ACA​GTG​GAA​ATG​TTA​CC-3′ and reverse 5′-GGT​AAC​ATT​TCC​ACT​GTC​ACA​AAA​ACA​CCT​GGA​TAG​AG-3′. Both constructs were transformed into *E. coli* ZYCY10P3S2T cells. For minicircle production: transformed cells underwent primary culture (3 mL LB + 50 μg/mL kanamycin, 30 °C/250 rpm, 3 h); secondary culture volume was calculated as [0.0002/OD_600_] × desired volume before inoculation into 100 mL TB/kanamycin (30 °C/250 rpm, 16 h); induction used 100 mL LB + 4 mL 1N NaOH +0.12 mL 20% L-arabinose (30 °C/250 rpm, 3 h → 37 °C/250 rpm, 2 h); cells were harvested (6,000 × g, 12 min, 4 °C); and minicircle DNA was purified with QIAGEN Plasmid Plus Maxi Kit (QIAGEN, Hilden, Germany).

### Cell transfection

HEK 293FT cells (Thermo Fisher Scientific) were maintained in DMEM supplemented with 10% fetal bovine serum (FBS) at 37 °C/5% CO_2_. For transfection experiments, 5 × 10^5^ cells/well were seeded in 12-well plates and incubated for 20–22 h to reach 70%–80% confluency. Transfection complexes were prepared by combining 1 µg plasmid DNA with 3 µL TransiT-X2 reagent (Mirus Bio) in Opti-MEM, adjusted to 100 µL total volume, followed by 20-min room temperature incubation. The complexes were then added dropwise to cell cultures, plates were gently rocked for even distribution, and cells were returned to the 37 °C/5% CO_2_ incubator for 48 h prior to analysis.

### Intravenous tail injection

Mice were anesthetized with 2% isoflurane and secured in a tail-vein injection restrainer. A solution containing 60 µg plasmid DNA complexed with TransIT®-EE Hydrodynamic Delivery Solution (Mirus Bio, Madison, WI, USA) was prepared at a volume calculated by the formula: [mouse weight (g)/10] + 0.1 mL. The mixture was injected into the lateral tail vein using a 27-gauge needle over 4–7 s. Following injection, mice were immediately returned to their cages and monitored until full recovery from anesthesia.

### Activated partial thromboplastin time (aPTT) examination

Activated partial thromboplastin time (aPTT) was measured as previously described (Kao et al., 2021). Briefly, mice were anesthetized with 2% isoflurane, and blood was collected *via* retro-orbital sinus puncture. A 90 µL blood sample was immediately mixed with 10 µL 3.2% sodium citrate and incubated for 10 min at room temperature. Subsequently, 50 µL citrated blood was transferred to a cartridge and analyzed using the Coag Dx Analyzer (IDEXX, Westbrook, ME, USA). Clotting time was automatically recorded, with values exceeding 300 s truncated to 300 s per instrument protocol.

#### FVIII activity assay

Human FVIII activity was quantified using a chromogenic assay (Siemens, Marburg, Germany) as previously described (Kao et al., 2021). Briefly, plasma samples were diluted 1:31 in saline, then incubated with FIXa and FX reagents at 37 °C for 90 s. Substrate reagent (containing thrombin-specific chromophore) was added, followed by stopping buffer after 6 min. Absorbance at 405 nm was measured to calculate FVIII activity based on a standard curve.

#### Tail clip assay

Mice were anesthetized with 2% isoflurane and tails warmed in 37 °C saline for 2 min. A 3-mm distal segment was excised and immersed in 14 mL 37 °C saline for 15 min. After cauterization, the blood-saline mixture was weighed, centrifuged (500 × g, 10 min), and the pellet lysed with 6 mL RBC lysis buffer (10 min, RT). Hemoglobin concentration was quantified at 575 nm using 200 µL lysate.

#### Flow cytometry

Flow cytometry (BD Biosciences, Franklin Lakes, NJ, USA) quantified EGFP fluorescence in transfected HEK 293FT cells and recipient mouse livers. For cells, trypsinized suspensions were filtered through 40 µm mesh. For liver analysis, tissues were minced and digested in collagenase IV (0.5 mg/mL)/DNase I (0.05 mg/mL) in HBSS at 37 °C for 30 min, sequentially filtered through 70 μm and 40 µm meshes, treated with RBC lysis buffer, and single-cell suspensions were analyzed.

### Genomic DNA extraction and quantitative PCR (qPCR) analysis

Genomic DNA was extracted from 10–25 mg mouse liver tissue using the Presto™ DNA/RNA/Protein Kit (Geneaid). TaqMan probe-based qPCR was conducted using a QuantStudio 6 Pro system (Applied Biosystems) to detect the target transgene. This involved two primer sets: the hFVIII primer set, which includes a forward primer (ACC​AGC​ATG​TAT​GTG​AAG​GAG​TTC), a reverse primer (CCA​CAG​GTG​TGA​AGG​AGT​CTT​G), and a probe (AGCAGTCAAGATGGC); and the BDD FVIII primer set, consisting of a forward primer (CAT​GAC​CGC​CTT​ACT​GAA​GGT​T), a reverse primer (GGC​GTT​TCA​AGC​TTC​TTG​GT) and a probe (AGT​TGT​GAC​AAG​AAC​AC). Each reaction contained 10 μL of qPCRBIO SyGreen Blue Mix, 0.4 μL of each primer and probe, 20–60 ng of DNA, and water to reach a final volume of 20 μL. Cycling conditions were 95 °C for 3 min, then 40 cycles of 95 °C for 10 s and 60 °C for 1 min, followed by a melting curve from 60 °C to 95 °C at 0.1 °C/s.

### RNA extraction and droplet Digital-PCR (ddPCR) analysis

RNA was extracted from 10–25 mg liver tissue (post-flow cytometry) using the Presto™ DNA/RNA/Protein Kit (Geneaid Biotech Ltd., Taipei, Taiwan), followed by cDNA synthesis with Maxima™ H Minus Master Mix (Thermo Fisher Scientific). The cDNA samples were subjected to ddPCR analysis through a water-oil emulsion method on Bio-Rad’s QX200 system with 20 µL reactions containing 10 µL of ddPCR Supermix (#1863024), 1 µL of each primer and TaqMan probe, 1 µL of cDNA, and nuclease-free water. Cycling conditions were 95 °C/10 min; 40 cycles of 94 °C/15 s → 60 °C/1 min (2 °C/s ramp); 98 °C/10 min; 12 °C hold. Products were analyzed on the QX200 Reader (Bio-Rad) to obtain an absolute count of FVIII copies in 10,000 droplets.

### *In vivo* imaging system (IVIS)

Livers were collected post-sacrifice, washed with PBS, and placed in a Petri dish. Green fluorescent protein expression was detected using the SpectrumCT IVIS system (PerkinElmer, Waltham, MA, USA) with excitation and emission filters set at 465 nm and 520 nm, respectively.

### Immunofluorescent staining

Liver tissues were fixed in 4% paraformaldehyde (PFA), dehydrated in sucrose, and embedded in OCT compound (Leica, Wetzlar, Germany). Cryosections (10 µm) were prepared using a CM3050 S cryostat (Leica). Sections were permeabilized with 0.2% Triton X-100 for 15 min, blocked in 1% BSA/0.3 mM glycine/PBST for 1 h, then incubated overnight at 4 °C with primary antibodies: anti-FVIII (Merck Millipore, Burlington, MA, USA) and anti-E-cadherin (BD Biosciences, Franklin Lakes, NJ, USA). After washing, Alexa Fluor™ 488/647 secondaries (Thermo Fisher Scientific) were applied for 1 h. Nuclei were counterstained with DAPI (0.1 μg/mL), and slides were imaged using an FV3000 confocal microscope (Olympus, Tokyo, Japan).

### Hematoxylin and eosin (H&E) staining

Liver tissues were fixed in 10% neutral buffered formalin, dehydrated through graded ethanol series, embedded in paraffin, and sectioned at 4 µm. Sections were stained with hematoxylin and eosin (H&E) using standard protocols (Chen et al., 2024; Pu et al., 2024) and imaged using a ZEISS AXIO SCOPE.A1 microscope.

### Liver biochemistry analysis

Blood was collected into clot-activator tubes and incubated for 30 min at RT, then centrifuged at 10,000 × g for 1 min to obtain serum. Serum levels of ALT, AST, and ALKP were measured using the Catalyst One Veterinary Blood Chemistry Analyzer (IDEXX Laboratories, Westbrook, ME, USA) as described previously (Kao et al., 2023).

### Statistical analysis

Data are presented as mean ± SD or SEM. Statistical analyses used Student’s t-test, one-way ANOVA (Tukey *post hoc*), or Kruskal–Wallis test (Dunn *post hoc*), with significance thresholds at *p < 0.05, **p < 0.005, and ***p < 0.001.

## Results

### Minicircle vector preparation

**FIGURE 1:**
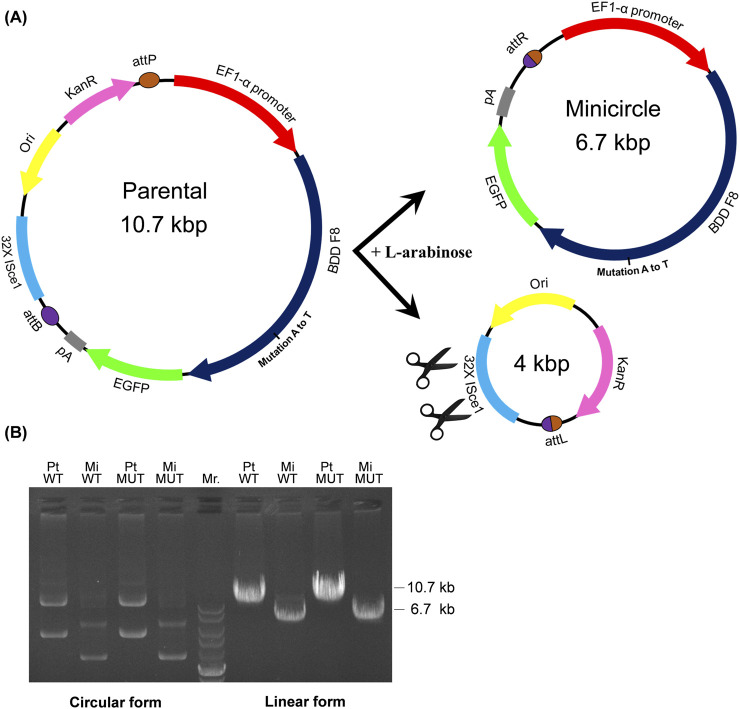
Minicircle FVIII plasmid production and validation. **(A)** Minicircle generation workflow: The FVIII transgene was cloned between *attB* and *attP* sites. L-arabinose induction initiated φC31 integrase-mediated recombination, separating the minicircle vector (containing FVIII) from the bacterial backbone. The backbone was degraded by *I-Sce*I endonuclease, enabling purification of the minicircle (∼4 kb). **(B)** Electrophoretic validation compared circular plasmids (*left*: parental open circular upper, supercoiled lower) to *Nhe*I-linearized DNA (*right*), confirming minicircle size reduction (∼4 kb vs. parental ∼8 kb) using a DNA ladder (Mr.: 10, 8, 6, 5, 4, 3, and 2 kb).

The minicircle DNA vector system reduced plasmid size through L-arabinose-induced recombination in *E. coli* ZYCY10P3S2T, excising the bacterial backbone while degrading it (Figure 1A). Electrophoresis confirmed a ∼4 kb size reduction in minicircle FVIII plasmids *versus* parental vectors (Figure 1B).

### Assessment of FVIII DNA transfection efficiency

**FIGURE 2:**
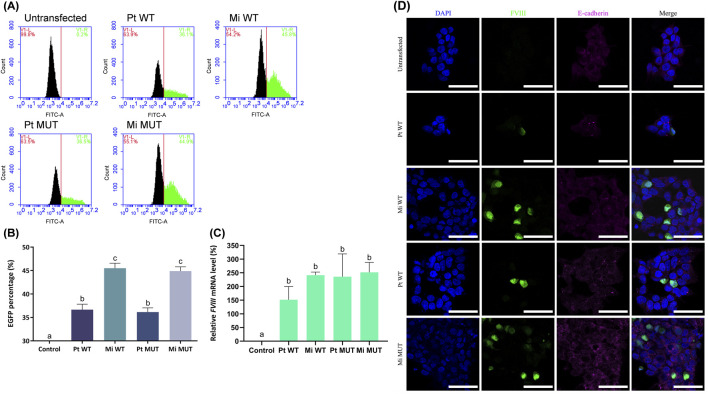
*In vitro* assessment of FVIII plasmid transfection efficiency. **(A)** Representative images illustrate flow cytometry quantification of EGFP^+^ HEK 293FT cells 24 h post-transfection. **(B)** Minicircle groups (Mi WT: 45.50% ± 1.08% and Mi MUT: 44.90% ± 0.90%, n = 3) showed significantly higher EGFP^+^ percentages *versus* parental plasmids (Pt WT: 36.67% ± 1.16% and Pt MUT: 36.13% ± 0.91%, n = 3). **(C)** FVIII mRNA levels (normalized to *GAPDH*, relative to Pt WT): untransfected (0.01% ± 0%), Pt WT (100%), Mi WT (151.39% ± 48.57%), Pt MUT (235.94% ± 83.41%), and Mi MUT (251.80% ± 36.33%; n = 3). **(D)** Confocal microscopy of transfected cells stained with DAPI (nuclei), anti-FVIII (red), and anti-E-cadherin (membrane; green). Scale bar: 50 µm (×1,000). Data: mean ± SD; compact letter display (CLD) denotes significance (*p* < 0.05, one-way ANOVA/Tukey).

HEK-293FT cells were employed for the assessment of transfection efficiency utilizing the following plasmid constructs: parental wild-type (Pt WT), minicircle wild-type (Mi WT), parental E1984V mutant (Pt MUT; Supplementary Figure S1), and minicircle E1984V mutant (Mi MUT). Flow cytometry revealed significantly higher EGFP^+^ cell percentages in minicircle groups (p < 0.001 vs. parental; Figures 2A,B), consistent with reduced plasmid size enhancing delivery efficiency. Quantitative analysis confirmed elevated FVIII RNA transcript levels in minicircle-transfected cells (Figure 2C). Confocal microscopy visualized robust EGFP expression in all transfected groups (with E-cadherin membrane/DAPI nuclear counterstains), while untransfected controls showed no signal (Figure 2D). These results demonstrate successful expression across all constructs, with minicircle vectors exhibiting superior transfection efficiency.

### Clotting restoration evaluation in FVIII KO mice

**FIGURE 3:**
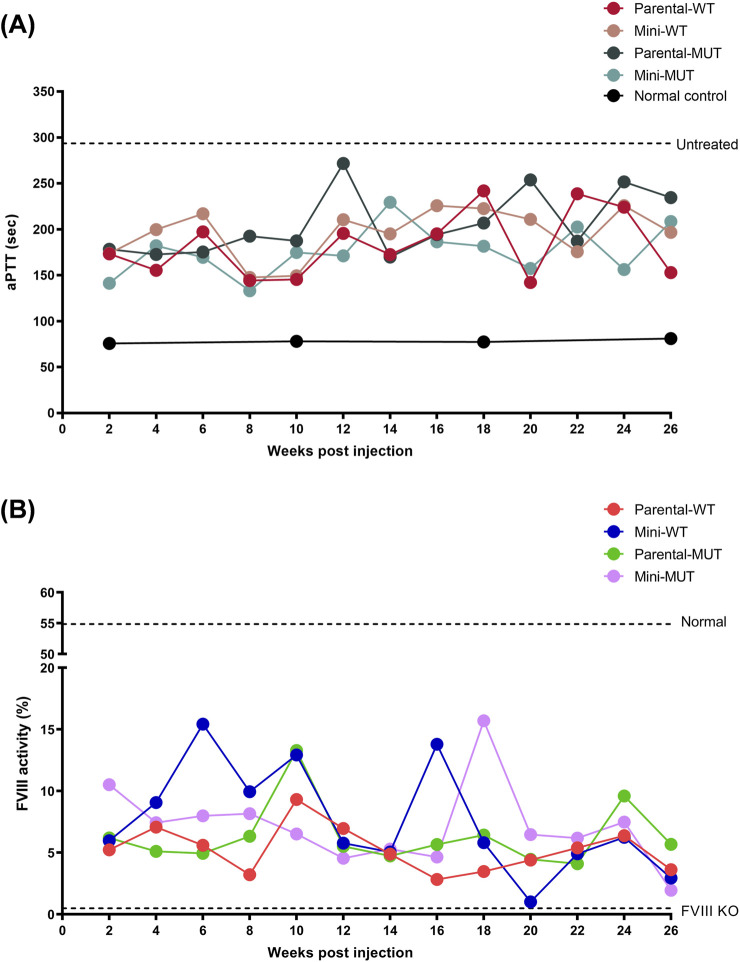
The assessment of coagulation function in hemophilia A mice throughout the therapeutic period. **(A)** aPTT dynamics. The aPTT was measured biweekly after intravenous tail injection of various FVIII DNA constructs. The dashed line indicates the baseline aPTT for those of untreated FVIII KO mice (293.57 ± 0.35 s; n = 7). **(B)** FVIII activity kinetics. The dashed lines denote the baselines activity levels of FVIII for normal controls (54.87% ± 1.93%; n = 4) and FVIII KO controls (0.49% ± 0.09%; n = 8), respectively. The data points illustrated here exclusively denote the mean values of each group for better clarity. The detailed experimental data and statistical outcomes are presented in Supplementary Tables S1–S4.

FVIII-KO mice were next used to evaluate the efficacy of intravenous delivery of FVIII plasmids (parental or minicircle, wild-type or mutant) over a 26-week period. As shown in Figure 3A, all untreated FVIII-KO mice exhibited activated partial thromboplastin time (aPTT) values exceeding 300 s, whereas normal control mice displayed values within the range of 50–100 s. Notably, all plasmid-treated mice, regardless of construct type, demonstrated aPTT values that consistently fell between those of untreated and normal control groups throughout the 26-week observation period (Figure 3A; Supplementary Tables S1, S2). Consistent with these findings, FVIII activity assays demonstrated that the control mice exhibited mean activity levels of approximately 55%, while FVIII-KO mice showed nearly undetectable activity (Figure 3B). In contrast, plasmid-treated groups dis-played FVIII activity within the intermediate range defined by control and untreated mice for the entire duration of the study, without significant differences among the various constructs (Figure 3B; Supplementary Tables S3, S4). Collectively, these results demonstrate that FVIII plasmid-based gene therapy effectively ameliorates coagulopathy in FVIII-KO mice and sustains functional efficacy for at least 26 weeks, although a gradual decline in activity was observed during the later phase of the study.

### Therapeutic durability and functional hemostasis assessment

**FIGURE 4:**
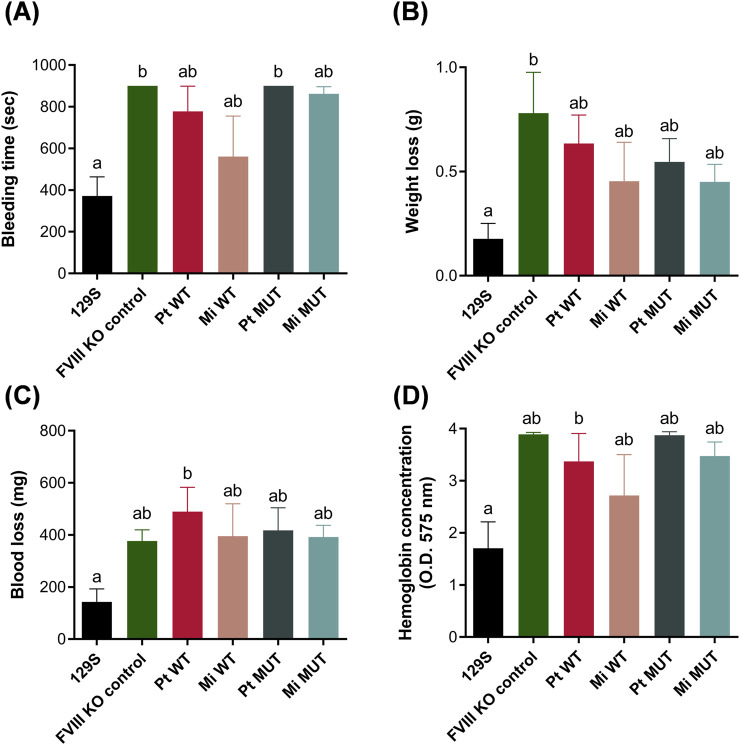
Tail clip assay for hemostatic function at 26 weeks post-treatment. Following tail transection (3 mm) and 900-s immersion in 37 °C saline: **(A)** Bleeding time: Wild-type (129S: 372.1 ± 91.31 s; n = 7) achieved hemostasis fastest, while FVIII KO (900 ± 0 s; n = 11) and Pt MUT (900 ± 0 s; n = 5) showed no clotting. **(B)** Weight loss: Minimal in 129S (0.18 ± 0.07 g; n = 7), highest in FVIII KO (0.78 ± 0.20 g; n = 5). **(C)** Blood loss: Wild-type (142.7 ± 50.12 mg; n = 7) had minimal loss, FVIII KO (377.0 ± 42.86 mg; n = 14) and Pt WT (489.5 ± 93.51 mg; n = 7) showed severe hemorrhage. **(D)** Hemoglobin: Wild-type (1.71 ± 0.51 g/dL; n = 7) lowest, FVIII KO (3.89 ± 0.04 g/dL; n = 9) and Pt MUT (3.88 ± 0.06 g/dL; n = 5) highest. Data: mean ± SEM. Statistics: Kruskal–Wallis/Dunn’s test (**A,C,D**; non-normal data) or ANOVA/Tukey **(B)**. Significance (*p* < 0.05) denoted by compact letter display.

While all constructs restored hemostatic function in recipient mice, the E1984V mutation did not markedly augment the therapeutic efficacy and durability of FVIII activities (Figures 3A,B), indicating that vector design—rather than transgene modification—governed sustained expression. To further validate the durability of FVIII gene therapies among different groups, tail clip assays (Supplementary Figure S2) were conducted at 26 weeks post-treatment. The results showed that upon the completion of a 26-week period, normal mice ceased bleeding within 6–7 min, while untreated FVIII-KO mice exhibited a complete inability to halt bleeding within the experimental duration of 15 min. The bleeding times of other groups receiving FVIII gene therapy were reduced to varying degrees in comparison to untreated FVIII-KO mice, along with clot formation in some recipient mice; however, no significant differences were noted among various treatment groups (Figure 4A). Body weight changes and blood loss volumes were consistent across groups, with the Mi MUT group exhibiting the least blood loss (Figures 4B,C). Hemoglobin levels in the collected blood samples showed no significant differences between groups, apart from the normal control (Figure 4D). These results demonstrate that the applied gene therapies retain a degree of hemostatic effectiveness 26 weeks after administration, highlighting the durability of single-dose therapies utilizing the presented minicircle FVIII gene constructs.

### Detection of FVIII DNA and its RNA transcripts in recipient liver

**FIGURE 5:**
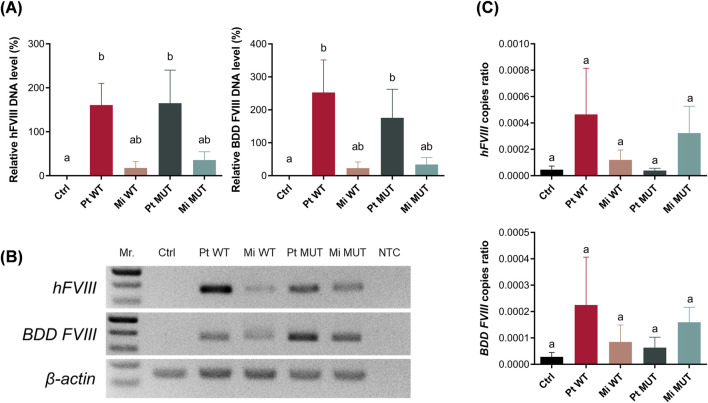
Detection of hFVIII transgene in recipient mouse livers. At the end of 26 weeks, genomic DNA was extracted from the liver tissue of recipient mice for TaqMan qPCR analyzing the existence of the hFVIII transgene with C2 domain-specific hFVIII and B-domain-spanning BDD-FVIII primer sets **(A)**. The results reveal higher hFVIII DNA levels in parental groups (Pt WT: 160.9% ± 49.13% hFVIII, 252.8% ± 98.83% BDD; Pt MUT: 165.2% ± 75.27% hFVIII, 175.9% ± 86.74% BDD) *versus* minicircles (Mi WT: 17.96% ± 14.48% hFVIII, 23.2% ± 18.53% BDD; Mi MUT: 35.88% ± 18.65% hFVIII, 34.32% ± 20.82% BDD; n = 5–7). **(B)** The representative electrophoresis images show the visualization of qPCR products from **(A)** (Mr.: marker; NTC: no-template control). **(C)** Droplet digital PCR analysis of hFVIII transgene expression post 26-week treatment. The results show detectable hFVIII transcripts across all treated groups (e.g., Mi MUT: 0.000324 ± 0.000203 hFVIII/β-actin; n = 5–7). Data are presented as mean ± SEM and analyzed by Kruskal–Wallis H test with Dunn’s *post hoc* test (*p* < 0.05; compact letter display).

At the end of the 26-week monitoring period, we examined the persistence of FVIII DNA and RNA transcripts in the liver tissues of recipient mice. TaqMan probe-based qPCR analysis revealed unexpectedly higher FVIII DNA levels in parental plasmid groups compared with minicircle groups (*p* < 0.001; Figures 5A,B). We hypothesize that the greater uptake efficiency of minicircle vectors may have elicited enhanced immune-mediated clearance, thereby reducing long-term DNA retention. In addition, the results of ddPCR analysis confirmed the presence of FVIII RNA transcripts across all treatment groups (Figure 5C), supporting the persistence of FVIII gene expression 26 weeks post-administration.

### Sustained hepatic FVIII expression

**FIGURE 6:**
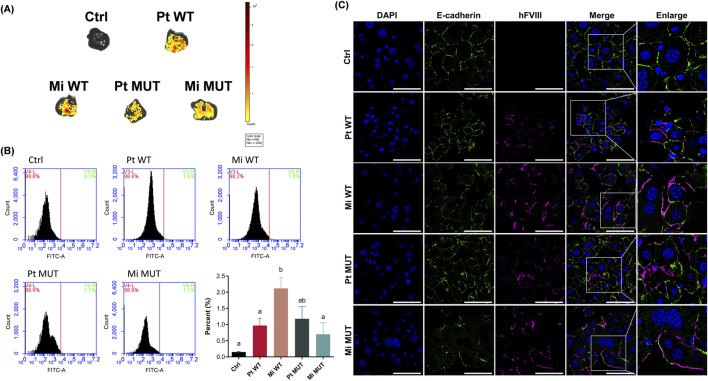
Hepatic FVIII protein expression at the end of 26 weeks. **(A)** IVIS imaging shows significant EGFP signals of EGFP in all recipient mice livers, contrasting with undetectable levels in untreated controls. **(B)** Flow cytometric analysis reveals EGFP-positive cell percentages in the hepatic tissues: untreated (0.15% ± 0.02%, n = 8), Pt WT (0.97% ± 0.23%, n = 7), Mi WT (2.12% ± 0.33%, n = 5), Pt MUT (1.18% ± 0.37%, n = 5), and Mi MUT (0.70% ± 0.36%, n = 6). **(C)** Confocal microscopy of liver sections stained with anti-FVIII and anti-E-cadherin antibodies confirms the localization of FVIII at the hepatocyte membrane, indicating functional secretion. Data are presented as mean ± SEM and analyzed by one-way ANOVA with Tukey’s *post hoc* test (*p* < 0.05; compact letter display). Original magnification is 1,000×; scale bars represent 50 µm.

The expression of EGFP encoded by vector constructs was detected in the livers of all recipient mice using *in vivo* imaging system (IVIS) analysis (Figure 6A). Correspondingly, flow cytometric evaluation of isolated liver cells revealed a significantly higher proportion of EGFP-positive cells relative to control groups (*p* < 0.001; Figure 6B). Confocal microscopy of liver sections stained with anti-FVIII and anti-E-cadherin antibodies showed FVIII localization at the hepatocyte membrane (Figure 6C). These findings demonstrate stable hepatic engraftment of FVIII DNA, enabling membrane-targeted protein expression and secretion for over 26 weeks.

### Hepatic safety assessment

**FIGURE 7:**
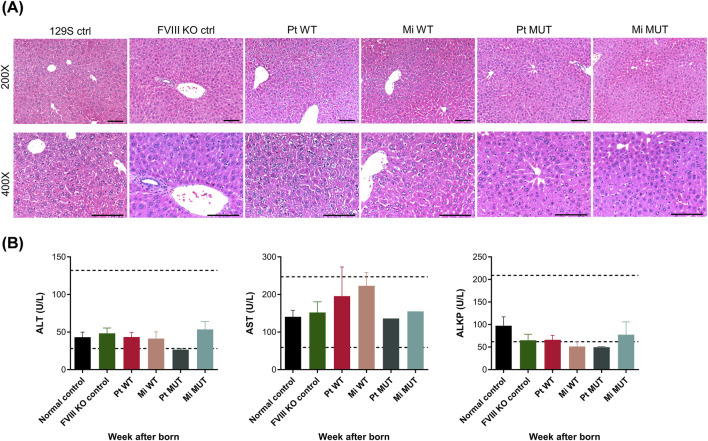
Hepatic safety assessment. **(A)** H&E staining of liver sections revealed normal hepatocyte morphology across all treatment groups, with no evidence of necrosis, inflammation, or structural damage compared to untreated controls. **(B)** Serum biochemistry analysis confirmed liver enzyme levels within physiological ranges: ALT (28–132 U/L), AST (59–247 U/L), ALKP (62–209 U/L). No significant differences were observed between treated and untreated mice. Original magnifications: 200× and 400×; scale bars: 100 µm.

Hematoxylin and eosin (H&E) staining of liver sections revealed normal hepatocyte morphology across all treatment groups, with no evidence of inflammation, necrosis, or structural damage compared to untreated controls (Figure 7A). Serum biochemistry analysis (ALT, AST, ALKP) showed levels within normal physiological ranges (ALT: 20–40 U/L; AST: 50–100 U/L; ALKP: 50–150 U/L), with no significant differences between treated and untreated mice (Figure 7B). These results confirm the long-term safety of intravenous FVIII DNA delivery, demonstrating no hepatic toxicity or adverse effects.

## Discussion

This study demonstrates that minicircle DNA constructs encoding WT FVIII and E1984V variants achieve sustained expression in FVIII-KO mice through L-arabinose-induced backbone excision, reducing plasmid size by ∼4 kb and mitigating immunogenicity. However, three translational barriers emerged: (1) Scalability limitations—large-scale fermentation risks inconsistent recombination and residual bacterial DNA (Alves et al., 2021; Gaspar et al., 2015; Ventura et al., 2022); (2) Purification bottlenecks—clinical-grade size-exclusion chromatography increases costs (Alves et al., 2021; Mitdank et al., 2021); and (3) Durability-immunity tradeoff—enhanced minicircle uptake initially boosted transfection (Figures 2A,B) but accelerated immune-mediated DNA clearance by week 26 (Figures 5A,B). Notably, although the E1984V mutation has been reported to improve the functionality and stability of FVIII (Wakabayashi et al., 2008), the advantages pertaining to therapeutic efficacy and durability linked to this mutation were not apparent in the present study (Figures 3A,B), indicating that vector design—rather than transgene modification—dominates long-term efficacy. The absence of hepatotoxicity (Figure 7) supports clinical potential, but resolving scalability and immune evasion remains critical for translation.

To evaluate therapeutic efficacy, hydrodynamic tail vein injection delivered four FVIII DNA constructs (parental/minicircle × WT/E1984V) over 26 weeks. Unlike prior nanoparticle-delivered BDD-FVIII under albumin promoters—which sustained effects <1 month (Kao et al., 2021)—this study employed minicircle backboneless vectors with the E1984V half-life-extending mutation. While minicircles enhanced *in vitro* transfection (Figure 2), *in vivo* assessments (aPTT, FVIII activity, tail-clip; Figures 3 and 4) showed no intergroup differences. Critically, all treated groups exhibited clinically relevant coagulation improvement *versus* FVIII-KO controls, aligning with evidence that even modest FVIII elevation (5%–10%) reduces spontaneous bleeding and joint damage in patients (Peyvandi et al., 2016; Srivastava et al., 2013). Thus, despite diminishing durability, this approach holds translational value for mitigating hemorrhage severity.

Exogenous DNA persisted in recipient livers at 26 weeks, confirmed by IVIS, flow cytometry, PCR, and histology (Figures 5, 6). Contrary to expectations, parental plasmids showed higher FVIII DNA levels than minicircles (Figure 5), potentially due to enhanced minicircle uptake triggering stronger early immune responses that accelerated DNA clearance (Kutzler and Weiner, 2008; Endmann et al., 2010; Smith et al., 2020). This suggests a tradeoff: reduced minicircle size improves transfection efficiency but may increase immunogenicity, leading to faster degradation.

Hydrodynamic tail vein injection, while effective in murine models, is not clinically practical due to its large injection volume and risk of transient liver damage (Suda and Liu, 2007). Alternative strategies—such as liver-targeted lipid nanoparticles (LNPs) with optimized ionizable lipids (Cui et al., 2022; Kulkarni et al., 2017), non-infectious virus-like particles (VLPs) mimicking viral tropism (Bhat et al., 2022; Nooraei et al., 2021), and image-guided hepatic portal injections enhanced by microbubble-assisted ultrasound (Kharaziha et al., 2009; Yoshino et al., 2006; Stoller et al., 2015), offer improved delivery efficiency, greater safety, and enhanced hepatocyte transfection. These clinically adaptable approaches significantly increase the translational feasibility and potential for non-viral gene therapy in hemophilia.

To further enhance therapeutic efficacy, DNA sequence design can be optimized by employing codon optimization and intron inclusion tailored to murine or human codon usage bias, which improves mRNA translation efficiency (Ward et al., 2011). Incorporating scaffold/matrix attachment regions (S/MARs) may stabilize transcriptional activity by interacting with transcription factors or structural proteins such as SATB1 and HMGA, thereby forming transcriptionally active regions (Hagedorn et al., 2011; Heng et al., 2004; Simcikova et al., 2015). Additionally, nuclear localization signals (NLS) could facilitate DNA entry into the nucleus of non-dividing hepatocytes (Hebert, 2003; Zanta et al., 1999), while inclusion of the woodchuck hepatitis virus posttranscriptional regulatory element (WPRE) at the 3′ end of the FVIII gene may further enhance mRNA stability and boost FVIII expression (Hebert, 2003; Klein et al., 2006). These combined strategies could substantially improve the performance and durability of non-viral gene therapy.

The administration of exogenous FVIII carries inherent immunogenicity risks, particularly in severe hemophilia patients where anti-FVIII antibodies can neutralize therapeutic efficacy (Lavigne-Lissalde et al., 2005). While the E1984V mutation was employed to extend half-life and enhance activity, its potential to alter epitopes may unpredictably modulate immune responses compared to wild-type FVIII. To preemptively address this, artificial intelligence (AI)-driven epitope mapping (e.g., using NetMHCIIpan or BepiPred-3.0 algorithms) could identify and modify immunodominant regions while preserving FVIII’s structural stability and coagulant function (Jurtz et al., 2017). For instance, computational redesign of FVIII A2/C2 domains has reduced antibody binding by 70% in murine models without compromising specific activity (Zakas et al., 2013). Integrating such deimmunization strategies during vector design may mitigate immune responses while extending treatment durability.

Although viral gene therapies can provide sustained transgene expression, they risk immune activation and inflammation due to viral components (Shirley et al., 2020; Zhou et al., 2004), which may compromise therapeutic efficacy and safety. In contrast, non-viral minicircle vectors avoid many of these issues by eliminating immunogenic bacterial elements such as the origin of replication (ORI) and antibiotic resistance genes (Vandermeulen et al., 2011; Schleef et al., 2015), thereby reducing innate immune recognition and inflammatory responses. As a result, minicircle-based gene therapy represents a promising, low-immunogenicity approach for hemophilia A, with the potential to improve clinical outcomes while overcoming the limitations associated with viral delivery systems.

## Conclusion

In this study, we evaluated the therapeutic efficacy of minicircle DNA constructs encoding the wild-type and E1984V variants of FVIII as a non-viral gene therapy strategy for hemophilia A management. *In vitro*, removal of the bacterial backbone during minicircle DNA generation led to significantly enhanced transfection efficiency in HEK 293FT cells. For *in vivo* evaluation, hydrodynamic tail vein injection of minicircle DNA into hemophilia A mice sustained the correction of coagulation deficits for at least 26 weeks. FVIII DNA and RNA transcripts remained detectable in hepatic tissues at 26 weeks post-treatment. Importantly, no evidence of liver toxicity was observed following therapy administration. These findings indicate that minicircle DNA-based gene therapy offers a promising novel approach for hemophilia A, with potential to improve patient outcomes and quality of life.

## Data Availability

The original contributions presented in the study are included in the article/Supplementary Material, further inquiries can be directed to the corresponding author.
