# Supplementary material for: Non-viral gene therapy for hemophilia A: long-term outcomes of minicircle FVIII delivery in a mouse model
Source: Front Pharmacol. 2026 Apr 9;17:1742144. doi: 10.3389/fphar.2026.1742144 (PMC13102653; doi:10.3389/fphar.2026.1742144)
Supplement: Supplementary file 1 [file DataSheet1.pdf]

SUPPLEMENTARY DATA

Supplementary Table S1. Activated partial thromboplastin time (aPTT, seconds) measured biweekly over a 26-week experimental period (mean ± SEM; N)

	Parental-WT			Mini-WT			Parental-MUT			Mini-MUT		
week	Mean	SEM	N	Mean	SEM	N	Mean	SEM	N	Mean	SEM	N
2	173.20	22.14	10	173.57	26.94	7	178.22	31.96	9	141.25	24.69	8
4	155.40	18.76	10	199.71	31.82	7	172.56	26.45	9	182.00	30.66	8
6	197.10	29.44	10	216.86	26.69	7	175.22	32.45	9	169.38	27.55	8
8	144.20	21.81	10	147.57	19.61	7	192.50	31.69	8	133.13	10.24	8
10	145.33	24.29	9	149.33	26.18	6	187.43	30.62	7	174.75	31.41	8
12	195.50	28.83	8	210.33	27.85	6	271.67	13.15	6	171.13	34.52	8
14	172.50	18.27	8	195.00	29.65	6	169.83	41.52	6	229.38	31.47	8
16	195.00	33.05	7	225.67	22.05	6	194.17	37.85	6	186.33	34.80	6
18	241.71	28.16	7	222.50	35.95	6	206.83	35.01	6	181.50	39.51	6
20	142.00	31.01	7	210.67	28.94	6	253.67	38.00	6	157.33	31.73	6
22	238.57	29.55	7	175.50	37.29	6	187.00	36.73	6	202.40	47.62	5
24	224.00	23.32	7	225.67	29.61	6	251.60	48.40	5	156.20	37.28	5
26	152.86	28.35	7	196.60	49.68	5	234.40	40.88	5	208.40	33.54	5

Supplementary Table S2. Statistical analysis of aPTT values using the Kruskal-Wallis H test with Dunn's post hoc test

Week /Group	2	4	6	8	10	12	14	16	18	20	22	24	26
Control	a	a	a	a	a	a	a	a	a	a	a	a	a
F8_KO	b	b	b	b	b	b	b	b	b	b	b	b	b
Pt_WT	ab	a	ab	a	a	ab	ab	ab	ab	a	ab	ab	a
Mi_WT	ab	ab	ab	ab	ab	ab	ab	ab	ab	ab	ab	ab	ab
Pt_MUT	ab	ab	ab	ab	ab	ab	ab	ab	ab	ab	ab	ab	ab
Mi_MUT	a	ab	ab	a	ab	a	ab	ab	ab	ab	ab	ab	ab

a, b: Within the same column, groups labeled with different letters indicate statistically significant differences ($p < 0.05$).

Supplementary Table S3. FVIII activity (mean ± SEM; N) measured over 26 weeks

	Parental-WT			Mini-WT			Parental-MUT			Mini-MUT		
week	Mean	SEM	N	Mean	SEM	N	Mean	SEM	N	Mean	SEM	N
2	5.23	1.86	10	5.97	2.52	8	6.20	1.47	9	10.52	2.87	8
4	7.08	2.00	10	9.07	2.89	8	5.10	1.57	9	7.43	1.31	8
6	5.60	1.60	10	15.43	11.66	7	4.94	1.37	9	7.99	2.18	8
8	3.22	1.34	10	9.95	3.81	8	6.34	1.50	8	8.16	2.48	8
10	9.32	4.62	9	12.93	6.57	7	13.29	5.96	7	6.51	1.25	8
12	6.97	3.14	8	5.77	2.29	7	5.51	1.71	7	4.55	1.05	8
14	4.88	2.08	8	5.06	2.90	7	4.74	2.41	6	5.29	1.59	8
16	2.83	0.67	7	13.80	6.89	7	5.66	3.14	6	4.64	1.62	6
18	3.47	2.26	7	5.81	2.42	7	6.43	3.51	6	15.70	9.95	6
20	4.39	1.40	7	1.01	0.71	7	4.46	1.07	6	6.46	2.80	6
22	5.39	2.18	7	4.91	1.85	7	4.10	2.52	5	6.19	2.53	5
24	6.38	2.95	7	6.25	1.64	7	9.60	4.56	4	7.48	2.64	5
26	3.62	1.70	7	2.93	1.32	6	5.67	1.46	4	1.95	1.17	5

Supplementary Table S4. Statistical analysis of FVIII activity using the Kruskal-Wallis H test with Dunn's post hoc test

Week /Group	2	4	6	8	10	12	14	16	18	20	22	24	26
Control	a	a	a	a	a	a	a	a	a	a	a	a	a
F8_KO	b	b	b	b	b	b	b	b	b	b	b	b	b
Pt_WT	b	abc	ab	b	ab	ab	ab	ab	bc	ab	ab	ab	ab
Mi_WT	ab	ac	ab	ab	ab	ab	ab	a	abc	b	ab	ab	ab
Pt_MUT	ab	bc	ab	ab	ab	ab	ab	ab	abc	ab	ab	ab	ab
Mi_MUT	ab	abc	ab	ab	ab	ab	ab	ab	ac	ab	ab	ab	ab

a, b, c: Within the same column, groups labeled with different letters indicate statistically significant differences ($p < 0.05$).

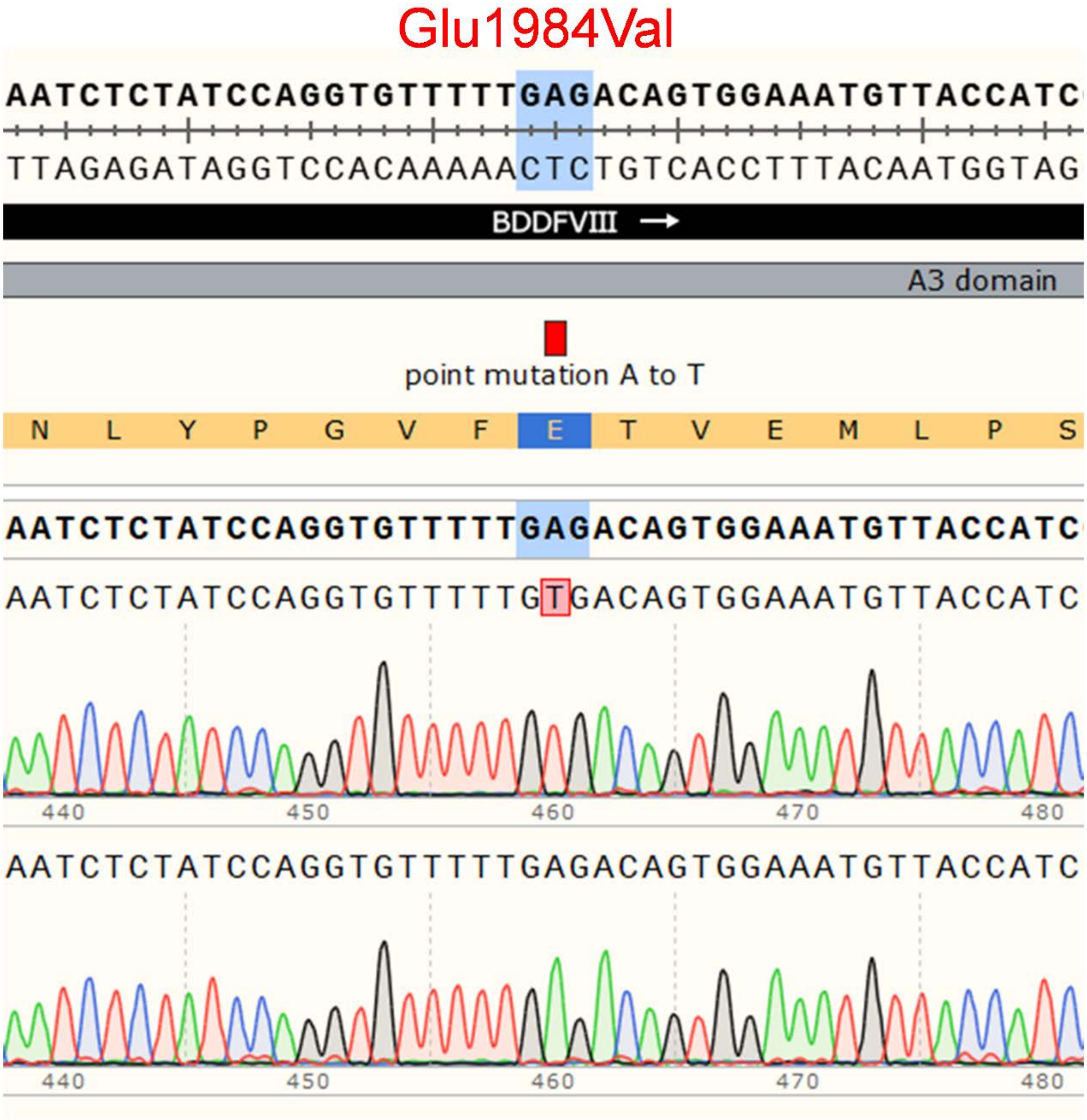

Supplementary Figure S1. Construction and verification of FVIII E1984V parental and minicircle plasmids. The 1984th glutamate codon (GAG) in BDD FVIII was substituted with valine (GTG) via site-directed mutagenesis PCR and confirmed by Sanger sequencing.

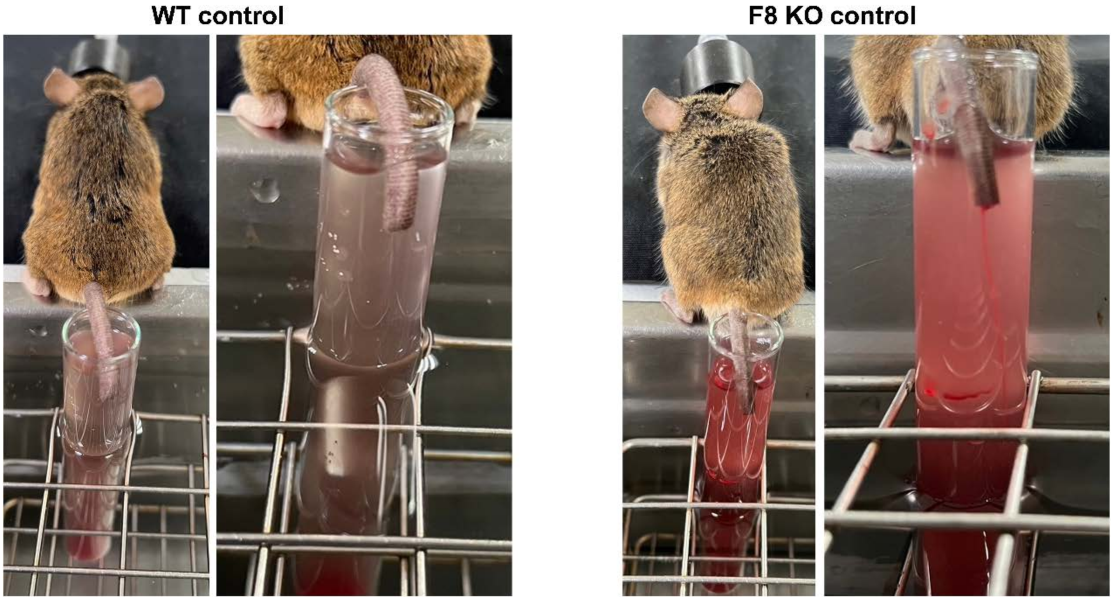

Supplementary Figure S2. Schematic of tail clip assay. After anesthesia, a 3-mm segment was excised from the distal tail and immersed in 37°C saline (0.9% NaCl) for 15 min. Wild-type controls achieved hemostasis within this period, while FVIII knockout (KO) mice exhibited persistent bleeding due to coagulation deficiency.
